# *GBA*-AAV mitigates sleep disruptions and motor deficits in mice with REM sleep behavior disorder

**DOI:** 10.1038/s41531-024-00756-5

**Published:** 2024-08-02

**Authors:** Ying Chen, Wei-Ye Xie, Dong Xia, Mu-Tian Zhang, Yan-Rui Sun, Wen-Xiang Duan, Yun Shen, Fen Wang, Wei-Min Qu, Zhi-Li Huang, Chun-Feng Liu

**Affiliations:** 1 Department of Neurology and Clinical Research Center of Neurological Disease, The Second Affiliated Hospital of Soochow University, 215004 Suzhou, China; 2 Jiangsu Key Laboratory of Neuropsychiatric Diseases and Institute of Neuroscience, Soochow University, 215123 Suzhou, Jiangsu China; 3 Department of Pharmacology, School of Basic Medical Sciences; State Key Laboratory of Medical Neurobiology and MOE Frontiers Center for Brain Science, Institutes of Brain Science, Fudan University, 200032 Shanghai, China; 4 Department of Neurology, Xiongan Xuanwu Hospital, 071700 Xiongan, China

**Keywords:** Circadian rhythms and sleep, Parkinson's disease

## Abstract

Sleep disturbances, including rapid eye movement sleep behavior disorder (RBD), excessive daytime sleepiness, and insomnia, are common non-motor manifestations of Parkinson’s disease (PD). Little is known about the underlying mechanisms, partly due to the inability of current rodent models to adequately mimic the human PD sleep phenotype. Clinically, increasing studies have reported that variants of the glucocerebrosidase gene (*GBA*) increase the risk of PD. Here, we developed a mouse model characterized by sleep–wakefulness by injecting α-synuclein preformed fibronectin (PFF) into the sublaterodorsal tegmental nucleus (SLD) of *GBA* L444P mutant mice and investigated the role of the *GBA* L444P variant in the transition from rapid eye movement sleep behavior disorder to PD. Initially, we analyzed spectral correlates of REM and NREM sleep in *GBA* L444P mutant mice. Importantly, EEG power spectral analysis revealed that *GBA* L444P mutation mice exhibited reduced delta power during non-rapid eye movement (NREM) sleep and increased theta power (8.2–10 Hz) in active rapid eye movement (REM) sleep phases. Our study revealed that *GBA* L444P-mutant mice, after receiving PFF injections, exhibited increased sleep fragmentation, significant motor and cognitive dysfunctions, and loss of dopaminergic neurons in the substantia nigra. Furthermore, the over-expression of *GBA*-AAV partially improved these sleep disturbances and motor and cognitive impairments. In conclusion, we present the initial evidence that the *GBA* L444P mutant mouse serves as an essential tool in understanding the complex sleep disturbances associated with PD. This model further provides insights into potential therapeutic approaches, particularly concerning α-synuclein accumulation and its subsequent pathological consequences.

## Introduction

Parkinson’s disease (PD) is a progressive neurodegenerative disorder marked by distinctive motor abnormalities that lead to considerable disability and morbidity in the late stages. PD is additionally linked to sleep abnormalities, such as rapid eye movement sleep behavior disorder (RBD), sleep fragmentation, insomnia, and excessive daytime sleepiness^1,2^. However, the precise mechanisms underlying these phenomena are not fully understood, partly due to the limitations of current mouse models in replicating the sleep disorder observed in human PD patients. Increasing research evidence suggests that sleep disorders are a crucial factor to consider in the study of neurodegenerative diseases. Our recent study has revealed a bidirectional relationship between sleep disorders and neurodegenerative diseases^3^. For example, during a 10–15 years period, a majority of individuals diagnosed with idiopathic rapid eye movement sleep behavior disorder (iRBD) exhibit a conversion rate exceeding 80% towards developing PD, dementia with Lewy bodies (DLB), or multiple system atrophy (MSA)^4–6^. The presence of iRBD is currently the strongest predictor of dementia development in PD and is associated with faster progression of non-motor symptoms^7,8^. Patients diagnosed with iRBD may exhibit subtle sensory, motor, and cognitive impairments, along with constipation, prior to the onset of PD and other α-synucleinopathies^6^. Therefore, therapeutic interventions to prevent the transition from iRBD to PD could potentially become disease-modifying treatments for PD^9^.

Currently, the exact pathogenesis of RBD is still unclear, but it may be associated with abnormal aggregation of α-synuclein protein and the polymorphisms of PD-associated genes (*GBA*, *LRRK2*, *SNCA*)^10^. Krohn et al. reported the identification of *GBA* mutations in 9.5% of individuals diagnosed with iRBD, encompassing a total of 1061 cases. In cases of Lewy body dementia and Parkinson’s syndrome, there was a notable reduction in *GBA* protein expression observed in distinct brain regions (the temporal cortex and caudate nucleus), as well as in the peripheral blood^11^. Besides, *GBA* represents a significant risk gene among Chinese early-onset PD patients, with a mutation frequency of 10.22%. Notably, heterozygous *GBA* variants constituted the most prevalent genetic cause identified in familial late-onset PD patients^12^. Particularly, the incidence of the L444P mutation was markedly higher in China than that reported in Western countries, whereas the frequency of the N370S mutation was significantly lower. Clinically, *GBA* mutation carriers exhibited greater impairments in various domains, including olfactory dysfunction, clock-drawing tests, nonverbal memory, and executive function. *GBA* gene mutations increased the risk of iRBD to some extent, although more clinical samples were needed for verification. Patients with *GBA*-associated PD more frequently exhibit various non-motor symptoms, including dementia, neuropsychiatric disorders, and autonomic nervous dysfunctions^13^. Despite the increasing research on sleep disturbances related to PD, there was limited exploration of the sleep characteristics in *GBA* L444P-mutant mice. To address this issue, we studied the sleep–wake behavior of *GBA* L444P mutant mice following the administration of α-synuclein preformed fibronectin (PFF), aiming to mimic the clinical manifestations of RBD patients.

The *GBA* gene codes for the lysosomal enzyme glucose cerebrosidase (GCase), which is vital in regulating glycosphingolipid balance. Specifically, GCase facilitates the conversion of sphingolipid–glucose–ceramide (GlcCer) and glucose–sphingosine (GlcSph) into glucose combined with ceramide and sphingosine, respectively^14^. Notably, a hallmark of PD is the presence of neuronal protein inclusions called Lewy bodies, which are primarily composed of α-synuclein^15^. Mutation in *GBA* typically leads to reduced GCase enzyme activity, which in turn compromises the autophagy–lysosomal system. As a result of this disruption, there is a lipid imbalance due to the accumulation of GlcCer and GlcSph, further promoting α-synuclein aggregation^15^. Consequently, such intracellular lipid and protein build-ups induce endoplasmic reticulum stress, leading to mitochondrial damage^16^. Interestingly, the absence of a correlation between residual GCase activity and disease severity supported the hypothesis that additional modifier genes may contribute to the observed phenotypic variation^17^.

In this study, we delineated the nature and degree of sleep/wake alterations in a precursor RBD animal model of PD. Following the injection of PFF into the sublaterodorsal tegmental nucleus (SLD) of the *GBA* mutant mice, we faithfully recapitulated a range of motor and non-motor symptoms characteristic of the progression from RBD to Parkinsonian syndrome. Evidence of this was the progressive neurodegeneration of midbrain dopamine neurons, leading to motor impairments similar to those seen in PD patients^18^. Subsequently, we systematically analyzed the potential contributions of each regulatory process to these sleep/wake disturbances following *GBA*-AAV injection, offering preliminary insights into the mechanisms responsible for sleep alterations in *GBA* mutant mice.

## Results

### WT mice showed decreased REM sleep after five months of PFF injection

**Fig. 1:**
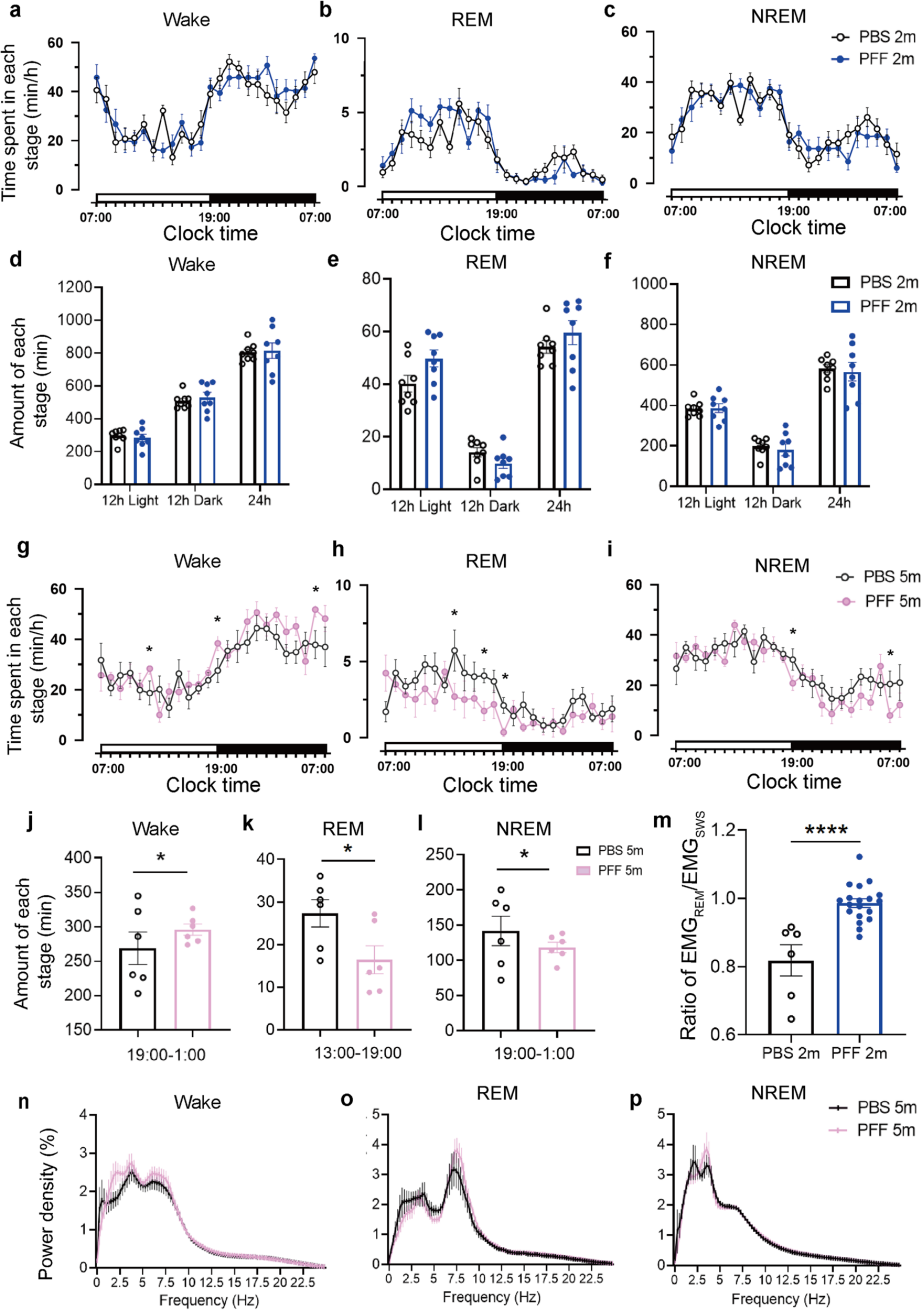
WT mice exhibited a decrease in REM sleep during the daytime at 5-month after PFF injection. **a–c** Time spent in each stage of Wake, REM, and NREM sleep after PFF infection at 2 months in WT mice over a 24 h light–dark cycle (*n* = 8). **d–f** Total amount of time in each stage, Wake, REM, and NREM states during the light and dark phases after PFF infection at 2 months in WT mice (*n* = 8). Statistical significance was determined using two-way ANOVA followed by Bonferroni’s multiple comparison test. **g–i** Time spent in each stage of Wake, REM, and NREM sleep after infection PFF at 5 months in WT mice over a 24 h light–dark cycle (*n* = 6). **j–l** Total amount of time in each stage, Wake, REM, and NREM states (*n* = 6). **m** Statistical analysis of the ratio of EMG_REM_/EMG_SWS_ across PBS/PFF administration at 2 months in WT mice. **n–p** EEG power spectrum of Wake, REM, and NREM sleep were both not altered after PFF injection of WT group mice (*n* = 6). Statistical significance was determined using the unpaired two-tailed Student’s *t*-test. All data are shown as mean ± SEM. *P < 0.05, ****P < 0.0001.

We initially injected PFF together with AAV2/9-hSyn-EGFP into the SLD region of mice. Two months following the PFF injection, we utilized synchronized video and EEG/EMG recordings to evaluate the emergence of RBD-like behaviors and alterations in the sleep–wake cycle. As shown in Fig. 1a–f, sleep analysis showed no significant change in total sleep duration and wakefulness after 2 months of PFF injection. A comprehensive analysis of EEG/EMG signals and corresponding video data indicated that PFF-induced neuronal damage in the SLD region elicited RBD-like symptoms in mice. Specifically, during REM sleep, we observed increased EMG activity after PFF injection (Fig. 1m). Video recordings captured subtle motor behaviors in mice, such as ear, head, and body tremors. More noticeable behaviors resembled dream enactment commonly seen in RBD patients, such as grasping, gnawing, or engaging in foraging-like activities during REM sleep (Supplementary Videos 1 and 2).

Following the PFF injection, no sleep disturbances were observed during the initial 2 months. However, extended observation at 5 months, we found that PFF group mice exhibited increased wakefulness (19:00–1:00), decreased NREM sleep (19:00–1:00), and reduced REM sleep (13:00–19:00) (Fig. 1j–l), while EEG parameters remained unchanged (Fig. 1n–p). Upon detailed examination of sleep architecture, as shown in Supplementary Fig. 2, there were an increase in the number of sleep–wake transitions. Notably, the number of micro-arousals (<16 s) increased significantly during both the light and dark phases. Additionally, there was an elevation in the average duration of wakefulness during the dark phase, suggesting sleep fragmentation.

### *GBA* L444P mutant mice exhibit no abnormalities in sleep architecture or spontaneous locomotor activity

**Fig. 2:**
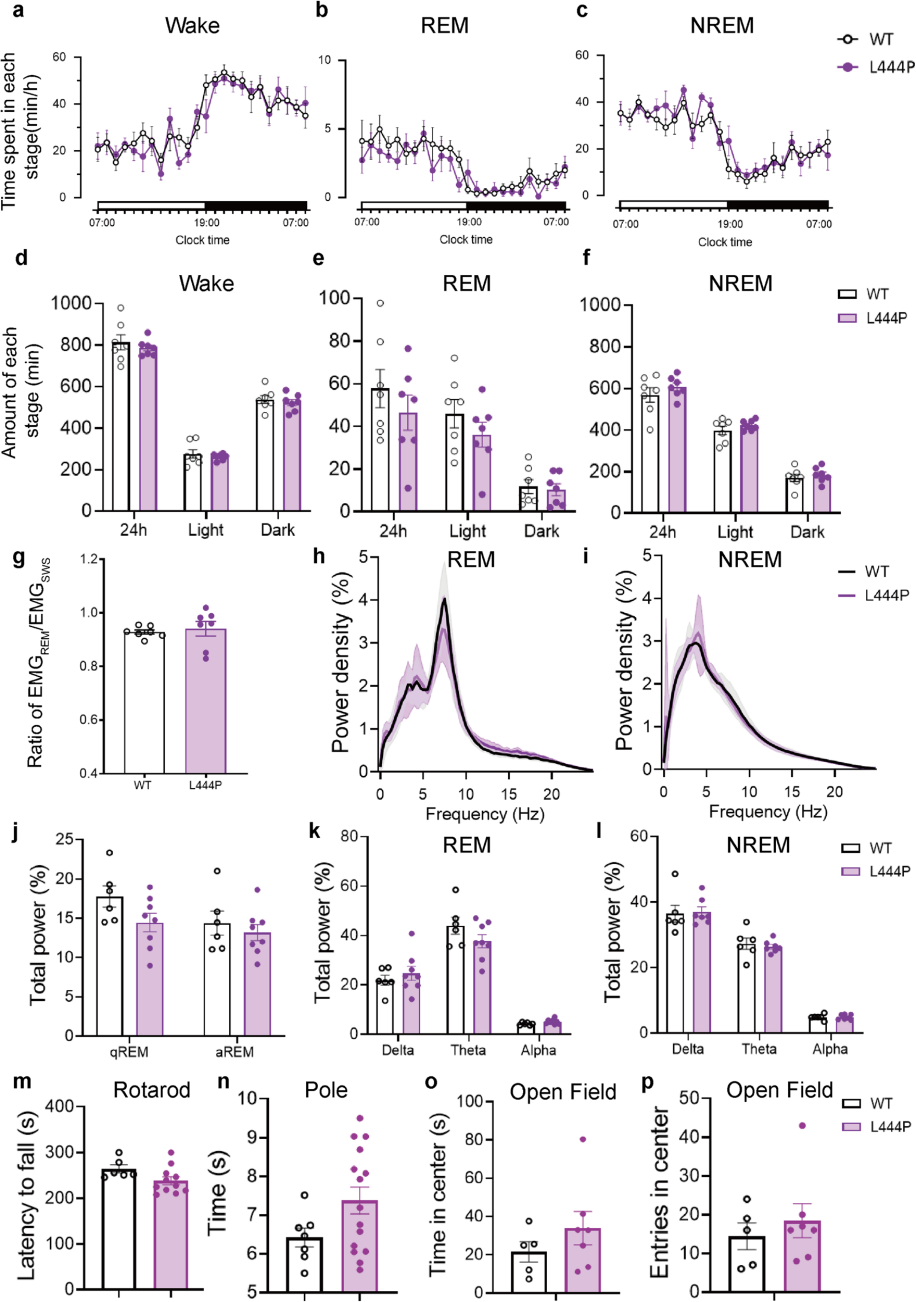
Evaluation of sleep architecture and motor behaviors in WT and L444P mutant mice. **a–c** Time spent in each stage of Wake, REM, and NREM sleep in WT and L444P mutant mice over a 24 h light–dark cycle (*n* = 7). **d–f** Total amount of time in each stage of Wake, REM, and NREM states during the light and dark phase (*n* = 7). **g** Statistical analysis of the ratio of EMG_REM_/EMG_SWS_ between WT or L444P mutant mice (*n* = 7). **h** and **i** EEG power spectrum of REM and NREM sleep were both not altered in L444P mice (*n* = 7) compared to WT mice (*n* = 6). **j–l** Statistical analysis of relative EEG power in WT (*n* = 6) and L444P mice (*n* = 7). Statistical significance was determined using two-way ANOVA followed by Bonferroni’s multiple comparison test. **m–p** The Rota-rod test, pole test, and open field test were performed between WT (*n* = 5–7) and L444P mice (*n* = 7–15). All data points represent the mean ± SEM. Statistical significance was determined using the unpaired two-tailed Student’s *t*-test.

We conducted EEG/EMG recordings and behavioral assessments to examine whether the GBA L444P mutation could directly induce early symptoms of PD in mice. As shown in Fig. 2a–f, there were no significant differences in the overall amount of sleep or wakefulness over 24 h period between the *GBA* L444P mutant mice and WT mice. Further analysis of EEG power during REM and NREM sleep revealed no significant variances in delta and theta waves (Fig. 2h–l). Since our study primarily focused on the RBD animal model, we performed a quantitative analysis of muscle tone during REM sleep in *GBA* L444P mutant mice. The results indicated that the *GBA* L444P mutation did not result in alterations in muscle tone during REM sleep (Fig. 2g). Subsequently, we evaluated the motor ability of the L444P mutant mice through rota-rod and pole climbing tests, which showed no motor disturbance (Fig. 2m, n). Furthermore, the open field test revealed that L444P mutant mice did not appear to have anxiety-like behavior (Fig. 2o, p). In summary, the L444P mutant mice did not exhibit abnormalities in sleep architecture or spontaneous locomotor activity.

### *GBA* L444P mutant mice showed increased arousal and decreased NREM sleep after two months of PFF injection

**Fig. 3:**
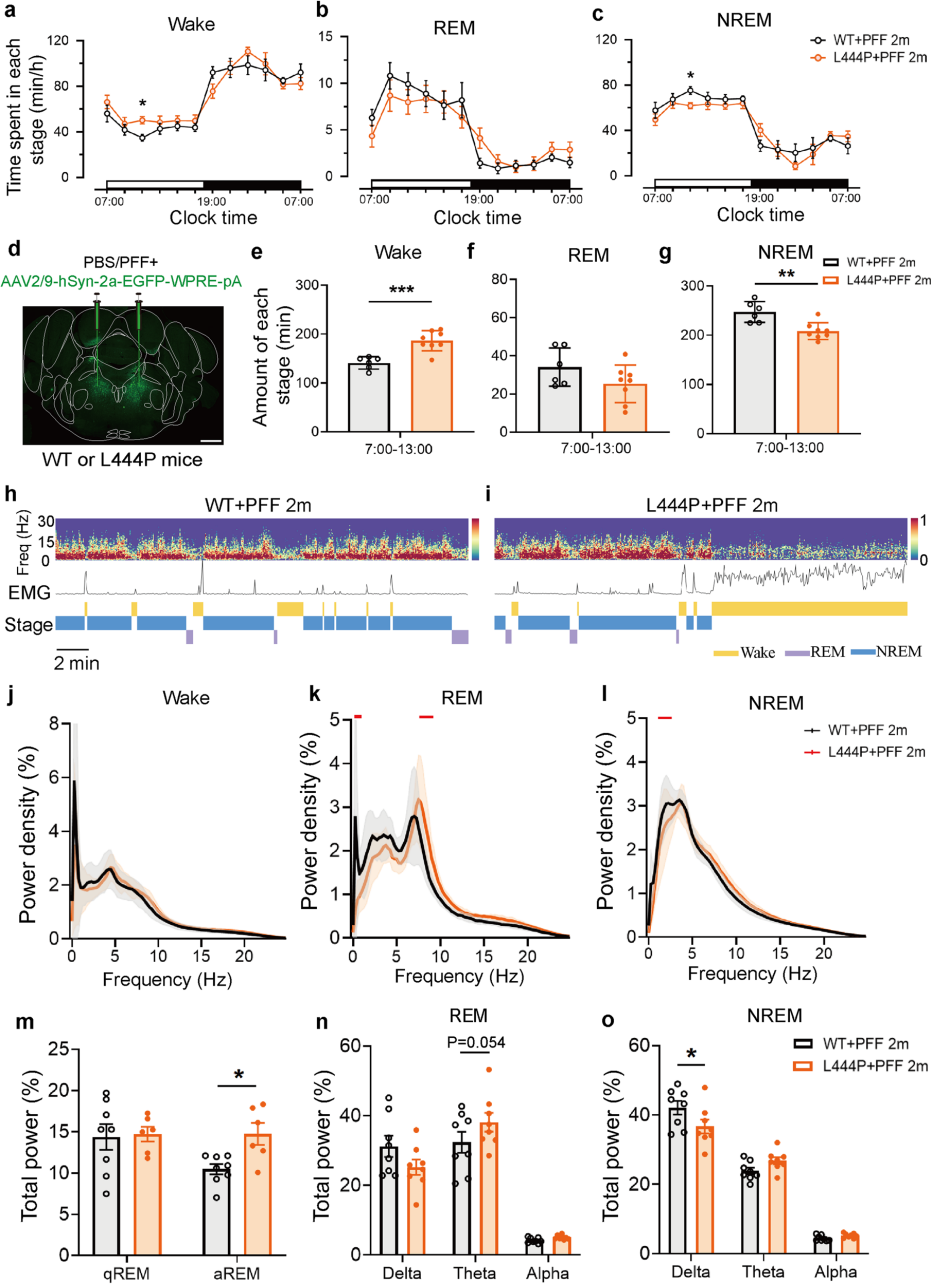
*GBA* L444P mutant mice showed increased arousal and decreased NREM sleep during the day after PFF injection. **a–c** Time spent in each stage of Wake, REM, and NREM sleep after infection PFF at 2 months in WT (*n* = 6) and *GBA* L444P mutant mice (*n* = 8) over a 24 h light–dark cycle. **d** Schematic of EGFP-AAV and PFF or PBS, a bilateral viral infection of SLD regions in young WT and *GBA* L444P mutant mice. Scale bars, 100 μm. **e–g** Total amount of time in each stage (7:00–13:00). (*n* = 6–8). Statistical significance was determined using the unpaired two-tailed Student’s *t*-test. **h** and **i** Typical EEG power spectrograms (light period, 14:00–14:30; top), EMG waveforms (middle), and Stage (bottom) of WT and L444P mutant mouse after PFF injection for 2 months. **j–l** EEG power spectrum of Wake was not altered, but REM and NREM sleep were both altered after PFF injection of L444P mice (*n* = 7) compared to WT mice (*n* = 8). Red dots indicate frequency bins that are significantly different between the two groups (*P* < 0.05, two-sided paired *t*-test with Bonferroni multiple comparison correction). **m–o** Normalized EEG power spectra in qREM and aREM. And aREM (8.2–10 Hz) was elevated in L444P mice compared to WT mice injected with PFF, but qREM (6.5–7.5 Hz) showed no difference between the two groups. During the REM sleep phase, theta was elevated in the L444P mice group (*P* = 0.054), and delta was decreased during the NREM sleep phase, suggesting a shallower sleep depth (*n* = 7–8). Statistical significance was determined using two-way ANOVA followed by Bonferroni’s multiple comparison test. All data are shown as mean ± SEM. *P < 0.05, **P < 0.01, ***P < 0.001.

Given that the *GBA* L444P mutant mice did not exhibit alterations in sleep duration, sleep structure, or muscle tone during REM sleep, we proceeded to observe the impact of *GBA* L444P mutation towards the PFF-based RBD model. We conducted long-term EEG/EMG recordings over a 24 h period. After 2 months of PFF injection, the *GBA* L444P mutant mice exhibited increased wakefulness and decreased NREM sleep (7:00–13:00) during the light phase compared to WT mice. This sleep disturbance, predominantly evident during the light phase, resembled the insomnia and sleep fragmentation seen in PD patients (Fig. 3a–g). Notably, the sleep/wake cycle of *GBA* L444P mutant mice exhibited greater sleep fragmentation, characterized by more frequent short wake episodes (<16 s) and increased transitions between NREM and wakefulness (Supplementary Fig. 4). In addition, the reduction in NREM sleep duration in *GBA* L444P mutant mice was consistent with sleep patterns observed in PD patients (Fig. 3g). Moreover, representative EEG, EMG, and EEG spectrograms were depicted after PBS and PFF injections (Fig. 3h, i). While the EEG power spectrum during wake remained unchanged, both REM and NREM sleep exhibited alterations in the L444P group compared to the WT group post-PFF injection (Fig. 3j–l). Spectral EEG analysis indicated increased aREM in the L444P mutant mice compared to the WT mice following PFF injection. The associated EEG power densities indicated heightened theta power during REM (*P* = 0.054) and reduced delta power during NREM (Fig. 3m–o). These results suggested that the *GBA* L444P mutation leads to increased arousal, decreased NREM sleep, and alterations in REM sleep intensity in the PFF-based RBD model.

### *GBA*-AAV over-expression promotes restoration of REM sleep after two months of PFF injection

Initially, we employed the immunofluorescence technique to co-stain neurons (NeuN), microglia (Iba1), and astrocytes (GFAP) markers with the GBA antibody, respectively. Our data indicated predominant *GBA* protein expression within neurons. Subsequently, we developed a *GBA*-AAV controlled by the human synapsin neuron promoter and administered it to the SLD region to achieve over-expression of GBA protein. After 2 months of AAV injection, we extracted the brain tissue for the GCase activity assay. Our results showed a significant increase in GCase activity, over four-fold, in the GBA-AAV group compared to the control group (EGFP-AAV). Furthermore, we assessed GCase activity across six groups of both WT and L444P mutant mice, which were injected with PBS, PFF, or PFF + *GBA*-AAV, respectively. Our findings indicated a pronounced reduction in GCase activity in L444P mutant mice compared to WT mice. However, GCase activity increased notably after injecting PFF + GBA-AAV into the SLD brain region (Supplementary Fig. 3). To confirm GBA-AAV over-expression, we analyzed GBA protein levels before and after the injection. Western blotting experiments revealed a decrease in GBA protein levels following PFF injection, which were partially restored after the *GBA*-AAV injection, as shown in Supplementary Fig. 6a, b. Correspondingly, immunofluorescence imagery of GBA supported these findings (Supplementary Fig. 6c).

**Fig. 4:**
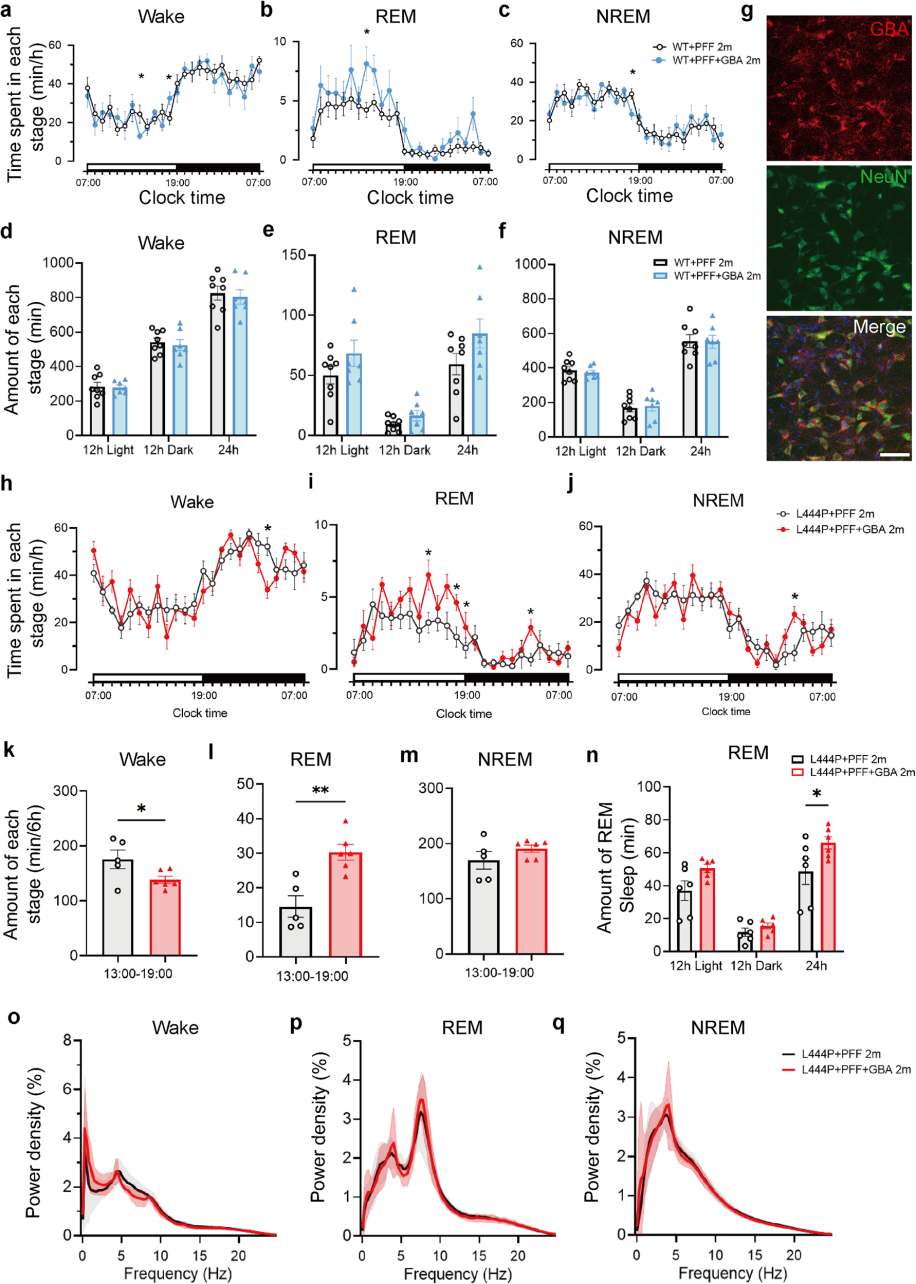
*GBA*-AAV over-expression promotes restoration of REM sleep in PFF-induced RBD mice. **a–c** Time spent in each stage of Wake, REM, and NREM sleep after infection PFF or PFF with *GBA*-AAV at 2 months over a 24 h light–dark cycle in WT mice (*n* = 8 vs. 7). **d–f** Total amount of time in each stage, Wake, REM, and NREM states. Statistical significance was determined using two-way ANOVA followed by Bonferroni’s multiple comparison test. **g** Immunofluorescent co-labeling of the *GBA* protein with the neuronal-specific marker NeuN. Scale bars, 50 μm. **h–j** Time spent in each stage of Wake, REM, and NREM sleep after PFF or PFF with *GBA*-AAV infection at 2 months over a 24 h light–dark cycle in *GBA* L444P mice (*n* = 7). **k–m** Amount of time in each stage, Wake, REM, and NREM states during the following 6 h (13:00–19:00). Statistical significance was determined using the unpaired two-tailed Student’s *t*-test. **n** Total amount of time in REM sleep during 24 h (*n* = 5–6). Statistical significance was determined using two-way ANOVA followed by Bonferroni’s multiple comparison test. **o–q** EEG power spectrum of Wake, REM, and NREM sleep were both not altered after *GBA*-AAV over-expression (*n* = 5–6). All data are shown as mean ± SEM. *P < 0.05, **P < 0.01.

To evaluate the effects of *GBA*-AAV over-expression on sleep patterns in the RBD model after 2 months, EEG/EMG analysis showed a slight increase in REM sleep after *GBA*-AAV injection compared with PFF injection alone in the WT mice, although this difference was not statistically significant (Fig. 4e). Notably, due to the homeodomain expression of the GBA protein in WT mice, *GBA*-AAV over-expression exhibited no significant effect (Fig. 4a–e). However, in *GBA* L444P mutant mice, *GBA*-AAV over-expression significantly increased REM sleep duration over a 24 h period (Fig. 4h–n). Specifically, there was a substantial increase in REM sleep during the light phase (13:00–19:00), which corresponds to the typical REM sleep period in the physiological state of mice (Fig. 4l). Furthermore, there was a concurrent reduction in arousal events during the light phase (13:00–19:00) (Fig. 4k). Nevertheless, no significant alterations were observed in the EEG parameters (Fig. 4o-q). As previously described, fragmented sleep was observed in *GBA* L444P mutant mice following PFF injection, but the GBA-AAV over-expression appeared to alleviate sleep fragmentation. Specifically, this was demonstrated by a longer mean duration of NREM sleep, a lower frequency of awakenings, a reduction in the number of micro-arousals and short segments of NREM sleep (Supplementary Fig. 7). These findings indicated that the GBA-AAV over-expression contributes to the restoration of REM sleep and maintenance of sleep states.

### *GBA*-AAV over-expression improved motor and cognitive performance and attenuated dopaminergic neuron loss after PFF injection

**Fig. 5:**
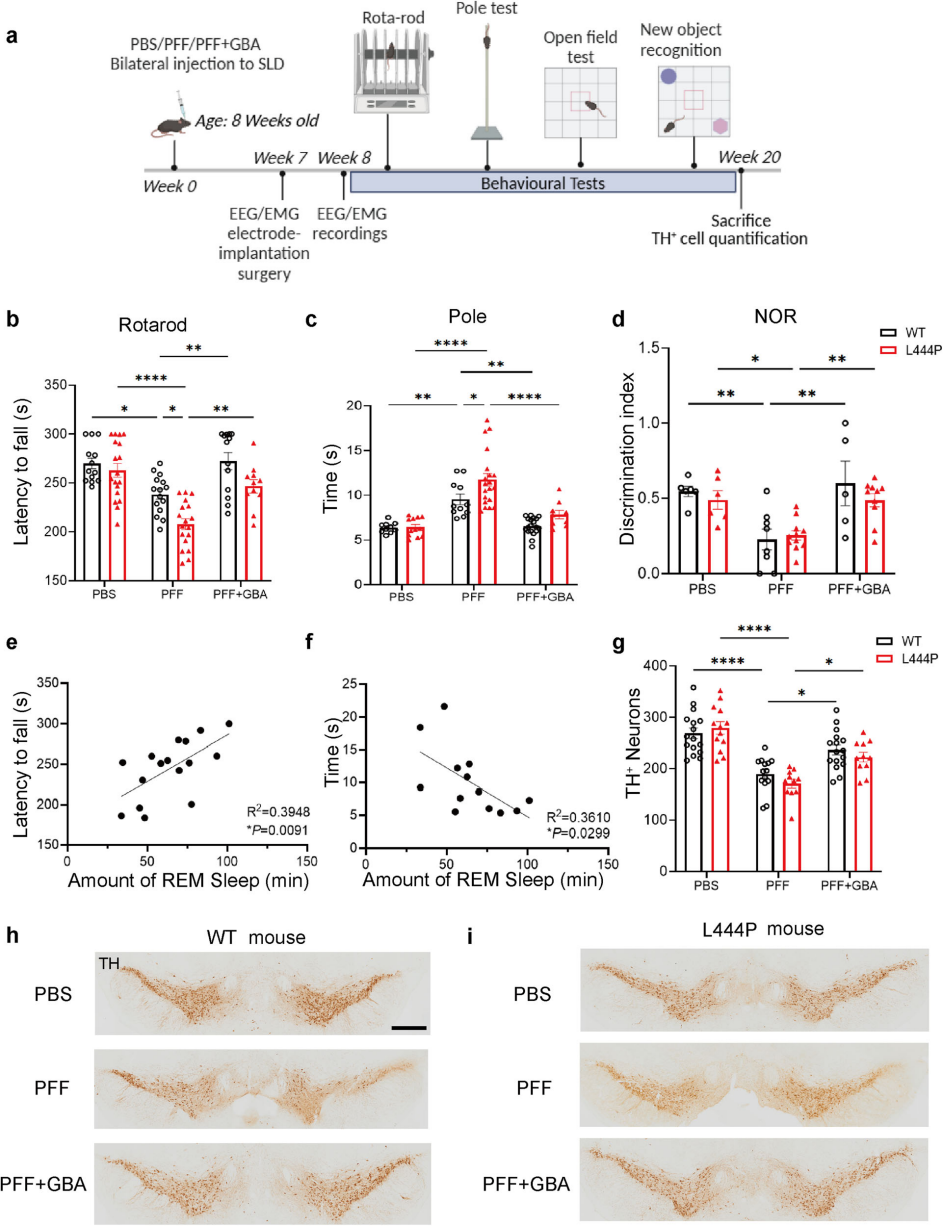
*GBA*-AAV over-expression restored REM sleep and ameliorated PFF-induced deficits in motor function, cognition, and nigral dopaminergic neuronal loss. **a** A schematic of the protocol and timeline used for the experiment. WT and *GBA* L444P mice were injected with PBS or PFF or PFF + *GBA* to induce the RBD animal model. **b** and **c** Assessment and comparison of the motor dysfunction (**b** Rota-rod test and **c** Pole test) between the PBS, PFF, and PFF + *GBA* mice groups after 2 months. **d** Effect of PFF plus *GBA*-AAV on the exploration of a novel and a familiar object in the retention trial in NOR test between different groups. **e** and **f** Correlation analysis between REM sleep volume and latency to fall in the rota-rod test or climbing time in the pole test. **g–i** Animals were sacrificed 5 months later and analyzed by immunohistochemistry (*n* > 10). Scale bars, 100 μm. Data are shown as mean ± SEM. Two-way ANOVA followed by Bonferroni’s post-tests. *P < 0.05, **P < 0.01, ****P < 0.0001.

In our study, we found that over-expression of *GBA*-AAV facilitated the recovery of REM sleep. To further explore its potential influence on motor and cognitive functions based on the PFF-induced RBD model, we performed rota-rod test, pole test, and new object recognition experiments. Figure 5a outlines our experimental protocol and timeline. After 2 months of PFF injection, we observed a decrease in rota-rod performance time and an increase in pole climbing time in WT mice, indicating motor dysfunction. In comparison, the *GBA* L444P mutant mice showed greater sensitivity to motor deficits in the rota-rod and pole test. However, following the administration of *GBA*-AAV to both WT and *GBA* L444P mutant mice, we noted significant motor function improvement compared to the PFF group (Fig. 5b, c). Additionally, our novel object recognition test results validated that *GBA*-AAV over-expression mitigated cognitive deficits induced by PFF administration (Fig. 5d). Our research suggested that *GBA*-AAV over-expression enhances REM sleep. Therefore, we hypothesized a potential connection between increased REM sleep and improved motor function. Our correlation analyses confirmed a positive association between REM sleep duration and Rota-rod performance, as well as a negative association with pole climbing latency (Fig. 5e, f). Taken together, these data implied a potential relationship between elevated REM sleep and enhanced motor function.

In addition to sleep and behavioral assessments, we conducted immunohistochemical evaluations of tyrosine hydroxylase-positive dopaminergic neurons after PFF administration in both WT and *GBA* L444P mutant mice (Fig. 5h, i). The quantitative analysis (Fig. 5g) indicated a 29.5% loss of dopaminergic neurons in WT mice following PFF injection. However, in the PFF + *GBA* group, this decline was limited to 12%. In the *GBA* L444P mutant mice, the loss of dopaminergic neurons reached 38.9% after PFF administration, whereas it was reduced to 20.3% in the PFF + *GBA* group compared to the PBS group. Overall, *GBA*-AAV over-expression appeared to ameliorate PFF-induced loss of dopaminergic neurons to some extent. Meanwhile, injection of PFF significantly caused the deposition of serine 129 phosphorylated α-synuclein (pS129 α-syn) in the L444P mutant mice, but GBA-AAV over-expression could alleviate the aggregation of pS129 α-syn (Supplementary Fig. 6d, e). Immunofluorescence images of pS129 α-syn were consistent with the above results (Supplementary Fig. 6f).

## Discussion

Our research revealed that the combination of the *GBA* L444P mutation with the PFF model led to alterations in sleep patterns reminiscent of the sleep disruptions observed in Parkinson’s disease patients, such as common insomnia and sleep fragmentation. These alterations were characterized by increased wakefulness during the light phase, reduced NREM sleep duration, augmented sleep fragmentation, shorter sleep bouts, and frequent transitions between wakefulness and NREM sleep. Additionally, the *GBA* D409V mutant mice exhibited a slight reduction in REM sleep, mirroring sleep features observed in PD patients^19^. These findings suggest that *GBA* L444P mutant mice manifest sleep patterns analogous to those in PD patients, offering a valuable animal model for studying sleep disturbances and further elucidating the underlying mechanisms associated with sleep-related symptoms in PD. The mechanisms underlying the heightened susceptibility of mice to PFF due to *GBA* L444P heterozygous mutations, and the reduced susceptibility via *GBA*-AAV over-expression remain unclear. However, this phenomenon may be linked to α-synuclein levels, as the neurotoxic effects on dopaminergic neurons induced by PFF necessitate the involvement of α-synuclein^20^. Initially, the interaction between GCase substrate and α-synuclein could precipitate an abnormal accumulation of α-synuclein^21^. Furthermore, diminished GCase activity in lysosomes could lead to an increased accumulation of glycosphingolipids and other lipid molecules, adversely impacting the composition of lipid membranes and thereby facilitating further aggregation of α-synuclein. Additionally, the misfolding of GCase proteins, along with subsequent endoplasmic reticulum stress, are key elements of this mechanism^22,23^. It is noteworthy that GCase deficiency is linked not only to endoplasmic reticulum stress, but also to oxidative stress, decreased ATP synthesis, and abnormal mitochondrial morphology. When compared to WT mice, GBA L444P mutant mice exhibit a significant loss of dopaminergic neurons and fibers in the striatum following MPTP treatment^24^. Moreover, the excessive accumulation of lipids or α-synuclein may initiate microglial activation, with extracellularly released α-synuclein directly engaging microglia to stimulate their activation^25^.

iRBD is widely regarded as an early clinical marker of α-synucleinopathies such as PD. However, the currently prevailing RBD animal models are not based on α-synucleinopathy^26,27^, thus lacking precise pathological representation of the transition from idiopathic iRBD to PD. As we know, the SLD region is a specific area within the brainstem that has been identified as playing a crucial role in the regulation of REM sleep^27,28^. Notably, the latest experimental studies have shown that in animal models, manipulative lesions or inactivation of the lateral olfactory tubercle neurons can induce RBD-like behaviors, suggesting their potential relevance in studying iRBD^27,29^. iRBD patients exhibit impairments in visuospatial abilities and executive function, which are commonly observed traits in α-synucleinopathies^30^. However, longitudinal studies suggest that a higher risk of conversion is linked more to impaired executive functions rather than visuospatial abilities^31,32^. The presence of a neurodegenerative process, coupled with cognitive decline, is indicated by cortical slowdown during wakefulness and REM sleep^33^. Despite increasing evidence, the identification of biomarkers remains a critical challenge in iRBD. Additionally, it has been reported that PD patients harboring GBA mutations exhibit higher incidences of cognitive impairment and neuropsychiatric symptoms, which may be associated with early cortical dysfunction^34,35^. Consistent with the cognitive impairments seen in clinical RBD patients, our findings also revealed that mice injected with exogenous PFF into the SLD nucleus for 2 months exhibited reduced exploration time towards novel objects in the NOR task, suggesting compromised learning and memory abilities.

Research on sleep architecture in PD patients predominantly reveals varied outcomes, with a consistent exception of diminished REM sleep atonia^36^. Noteworthy consistent observations include decreased sleep efficiency, enhanced sleep fragmentation, and elevated arousal episodes^37,38^. According to the classic two-process model of sleep regulation, the duration spent in NREM sleep is determined by the homeostatic sleep drive, which accumulates during wakefulness and dissipates during sleep^39,40^. Slow-wave activity (SWA), defined as the spectral power density of delta waves (0.75–4 Hz) during NREM sleep, is an effective indicator of sleep depth^41,42^. A groundbreaking study by Dan-Qian Liu’s team delineated two stages of REM sleep in mice: the quiet phase and the active phase. These stages exhibited markedly different facial expressions, autonomic nervous system activity, and brain electrical spectra. Despite the loss of skeletal muscle tone during REM sleep, the mice displayed considerable facial movements during the aREM sub-phase, akin to the observed staggered eye movements and facial muscle contractions during human REM sleep^43^. Interestingly, our study found distinctive electroencephalographic power spectra in *GBA* L444P-mutant mice following PFF injection compared to the WT mice. Specifically, there was an increase in the aREM spectral power during REM sleep and a decrease in the delta power during NREM sleep, indicating a reduction in sleep depth.

Although Cigdem et al. reported that *GBA* D409V mutations can reduce REM sleep^19^, the underlying mechanisms remain unclear. Our study, in contrast, focuses on the exogenous injection of PFF. We found that PFF-induced *GBA* L444P mutant mice experienced increased arousal and reduced sleep, so what are the potential neural and molecular mechanisms behind this? Several hypotheses have been suggested, such as the interaction between GCase substrates and α-synuclein, which may lead to α-synuclein accumulation, alterations in lysosomal membrane composition affecting autophagy and mitophagy, accumulation of misfolded GCase, endoplasmic reticulum stress, among others. Investigating these mechanisms in dopaminergic neuronal models implicated in RBD may offer new insights into these potential mechanisms.

In summary, our study reports the novel sleep phenotype observed in *GBA* L444P-mutant mice after injection of PFF into the SLD region. Our findings reveal that the *GBA* L444P mutation worsens existing sleep disturbances in the RBD model, leading to increased wakefulness, reduced overall sleep amounts, shallower sleep depth, and more fragmented sleep. Furthermore, *GBA*-AAV notably prevented the loss of dopamine neurons and rescued the behavioral deficits in the RBD model. Therefore, screening for iRBD in carriers of *GBA* mutations could potentially serve as an effective predictive measure for future development of α-synuclein-related diseases, although this requires further investigation through studies involving larger sample sizes.

## Methods

### Animals

Human *GBA* L444P mutant mice were obtained from the Jackson Laboratory (Stock No. 050598). The specific-pathogen-free (SPF) C57BL/6J male mice (8–10 weeks old, 20–23 g) were purchased from Shanghai Laboratory Animal Center, Chinese Academy of Science (SLAC, Shanghai, China). Mice were group-housed four to five per cage under a 12 h (7:00–19:00) light–dark cycle within a colony room at constant temperature (21 ± 0.5 °C) humidity (55 ± 5%). Food and water were provided ad libitum. Only male mice were used for experiments. All experimental protocols were approved by the Soochow University and Fudan University Animal Care and Use Committee.

### Preparation and characterization of preformed fibrils

We procured recombinant full-length human monomeric α-synuclein proteins from Proteos (RP-003) to create PFF. Following established protocols^44^, we created the PFF by continuously agitating α-synuclein monomers using an Eppendorf orbital thermomixer (1000 rpm, 37 °C) for 7 days. Subsequently, we assessed the endotoxin level (EU/ml) and confirmed it fell within permissible ranges (<0.5 EU/ml). After sonicating the mixture (2 s on/off, 300 s total, 15% amplitude), we measured the PFF concentration using a BCA protein assay and adjusted it to 5 mg/ml.

Next, we conducted a comprehensive analysis of the PFF using the Thioflavin T (ThT) assay and transmission electron microscopy (TEM, Tecnai G2 Spirit Biotwin) to characterize their fibrillar morphology via phosphotungstic acid negative staining. Previous studies have shown that sonication-induced fragmentation of PFFs increases their potential for nucleation and neuronal toxicity at certain length dimensions^45^. As demonstrated in Supplementary Fig. 1a, sonication significantly reduced the length of PFF fragments, with most fibrils being under 50 nm. In line with past research, such short fibrils are more likely to be taken up by neurons, leading to α-synuclein aggregation and propagation^46^.

### Stereotaxic injections

All surgical procedures were conducted within a sterile setting. The mice were anesthetized with a combination of isoflurane and oxygen (2–3%) and maintained at a constant body temperature using heating pads. Subsequent to scalp preparation and disinfection, the mice were positioned in a stereotaxic apparatus (RWD, Shenzhen, China). A small aperture (1 mm in diameter) was then carefully drilled above the target nucleus on the skull surface. Glass microtubules with a tip diameter of 30 mm, previously loaded with virus, sterile PBS, or PFF (200 nl PBS/PFF plus 10 nl AAV2/9-hSyn-EGFP site-reporting virus per injection), were progressively guided towards the bilateral SLD (AP/ML/DV: −5.20/±0.75/−4.10 mm). Subsequently, they were connected to an air-pressure propulsion system (Packhanni Fin) to administer the inoculum into the specified nucleus at an infusion rate of 5 nl/min. After the infusions, the pipettes were kept in situ for 10 min and then withdrawn slowly. Following the injections, the mice were sutured and caged individually to recover for 1 week. Following behavioral testing, the mice were euthanized to confirm viral expression.

### EEG/EMG electrode-implantation surgery

Briefly, the mice were anesthetized under a 1.5% isoflurane/oxygen mixture at a 0.6-LPM flow rate. After the mice were prepared and sterilized, and fixed in the stereotaxic head frame, two stainless-steel screws, which served as EEG electrodes, were implanted into the frontal (AP/ML: +1.50/–0.80 mm) and parietal (AP/ML: –1.50/–1.00 mm) bone surface. For EMG recordings, EMG electrodes were placed into the dorsal neck muscle to monitor muscular activity. All EEG electrodes were fixed to the skull via dental cement. Then, the mice were individually caged and allowed 7 days to recover before EEG/EMG recordings. Behavioral experiments were conducted 3 weeks later to allow for sufficient recovery and for viral expression.

### Video-polysomnography recording

Preceding the commencement of video-polysomnography recordings, the mice underwent a 48 h habituation period while connected to the sleep recording apparatus. The cortical EEG and nuchal EMG signals, sampled at a frequency of 512 Hz, were initially subjected to amplification and Biotex-based filtration. These processed signals were subsequently captured utilizing the CED 1401 digitizer in conjunction with the Spike 2 software (CED, UK). The transformed data from Spike 2 were subsequently translated into interpretable vigilance states through the application of the SleepSign software (Kissei Comtec, Japan). Importantly, the animal behavior monitoring system was synchronized with the polysomnography recording equipment for simultaneous observation.

### EEG spectral analysis

EEG recordings and analysis followed the previous protocols^47^. Cortical EEG and neck EMG signals underwent amplification and filtering (Biotex, Kyoto, Japan, EEG, 0.5–30 Hz). Using the fast Fourier transform (FFT) analysis module incorporated within the SleepSign software, we initially extracted EEG frequency spectral data. Subsequently, we employed MATLAB to convert the raw frequency data into an EEG spectrogram according to established EEG rhythms (delta: 0.65–4 Hz, theta: 6–10 Hz, alpha: 12–14 Hz, beta: 15–25 Hz)^48^. Hypnograms were concurrently exported to align with the EEG spectrogram. For sleep-stage analysis, EEGs and EMGs were automatically classified into wakefulness, REM sleep, and NREM sleep epochs using 4-s intervals, based on standard criteria.

### EMG signal quantification

Following established protocols^26,27^, MATLAB (Mathworks, USA) was employed to quantify nuchal muscle tone intensity. This quantification facilitated the determination of mean EMG values during REM and NREM episodes. To mitigate muscle tone alterations induced by vigilance stage transitions (e.g., NREM–REM–NREM or NREM–REM–Wake), we exclusively considered REM episodes lasting over 48 s, excluding the initial and final 8 s during quantification procedures.

### Rota-rod test

The Rota-rod test was used to evaluate Parkinsonian loco-motor dysfunction in mice. The test was conducted over 3 consecutive days. On Days 1 and 2, each mouse was trained to acclimate to the testing apparatus. The training procedure was as follows: the mice performed three trials of running on a continuously accelerating rod (4–40 rpm in 5 min), with a 10-min gap between each trial. On Day 3, the mice underwent the same protocol, and the duration (in seconds) until they fell off the rod was measured. The average duration across the three trials was calculated for each mouse.

### Open-field test (OFT)

Mice were gently placed in the middle of the field, and movement was recorded for 30 min using a video-tracking system. The amount of time spent in the central area of the arena, which was defined as a zone measuring 30 × 30 cm located at the center of the chamber, was measured.

### Pole test

To conduct the pole test, a wooden apparatus measuring 50 cm in length and featuring a 1 cm diameter pole was utilized. At the top of the pole, a ball with a diameter of 2.5 cm was positioned. During the test, the pole was elevated to a 90° angle relative to the ground. The mice were placed on top of the ball and allowed to descend spontaneously. The duration it took for them to reach the bottom from the top was recorded as the climbing time. Each mouse underwent five separate trials to calculate the average climb time.

### Novel object recognition (NOR) test

To evaluate the learning and memory abilities of mice, we employed the object location test (OLT), novel object recognition test (NORT) as previously described^49^. During the training phase, two identical objects were positioned at a distance of one-fourth of the total chamber width from each adjacent wall in a chamber measuring 35 × 35 × 25 cm. The mice were placed in the center of the chamber to explore freely for 5 min before returning to their cages for a 2 h rest. During the OLT testing phase, we moved one object diagonally to create a new object location. In the NORT testing phase, we replaced one object with a differently shaped new one. The mice were again placed in the center, and we recorded their behavior for 5 min using a video camera.

The recognition ability of mice towards novel objects or location was calculated as (time to explore novel object or location/total time to explore novel object or location and old one)×100%. Mice with significantly disparate exploration times between the two objects during the training phases of the NORT and OLT were excluded from the analysis.

### Buried food pellets test

To evaluate the olfactory abilities of mice, we conducted a buried food pellet test. Initially, the mice were subjected to 24 h of food deprivation with continuous access to water. On test day, they were placed in a clean cage (46 cm long, 24 cm wide, and 20 cm high) containing a 3 cm layer of fresh woodchips for 20 min. After acclimation, we replaced the bedding and buried a 1 g standard mouse chow pellet 1 cm deep. Then, the mice were introduced into the cage, and the time to locate the food pellet was recorded. The test had a 5-min limit, ending when the mice touched the pellet with their nose or forelimbs. After each trial, we cleaned the cage with ethanol and replaced the bedding to prevent any residual odor cues.

### Glucosylceramidase activity assay

To evaluate Glucosylceramidase (GCase) activity, we used Glucosylceramidase Activity Assay Kit (Fluorometric, ab273339). Initially, we prepared all necessary reagents, including 20 µM 4-methylumbelliferone standard (4-MU) solution, samples, and positive controls. Subsequently, we added 2–20 µl of the samples to a 96-well white clear bottom plate. For background control, we introduced an equivalent volume of assay buffer into designated wells. Following this, we prepared and introduced the Glucosylceramidase substrate into the appropriate wells. Fluorescence was then measured at 37 °C in end-point mode (Ex/Em = 360/445 nm). To conclude, we calculated Glucosylceramidase activity using a specific equation.

1 $${{GCase}\; {activity}}=\frac{B}{(30\times V\times P)}\times D={{{pmol}}}/{min }/{{{{mg}}}}=\mu {\rm {U}}/{{{mg}}}$$

*B* is the 4-MU amount from the standard curve (pmol), 30 is the reaction time (min), *V* is the sample volume added into the reaction well (ml), *P* is the Initial sample concentration in mg-protein/ml (mg/ml), and *D* is the dilution factor.

### Western blotting

For western blotting, the primary antibodies utilized were anti-GBA (ab55080; abcam), anti-α‐synuclein (phospho S129) (ab51253; abcam), β‐actin (A3854; Sigma). The SLD brain tissues were lysed in RIPA buffer (P0013B; Beyotime) with protease and phosphatase inhibitors (MCE) for 30 min and centrifuged at 12,000 rpm for 20 min at 4 °C. The supernatants were then collected, and protein concentrations were determined using the BCA Protein Assay Kit (23225; Thermo Scientific).

The samples were prepared with loading buffer (FD002; Fude), and 20 µg of protein per sample were separated on a 10% SDS–PAGE, then transferred to a polyvinylidene fluoride (PVDF) membrane at 100 V for 90 min. The membranes were blocked in TBST containing 5% non-fat milk for 1 h at room temperature and incubated overnight with primary antibodies at 4 °C. Following primary antibody incubation, the membrane was washed three times in TBST, then incubated with secondary antibody at a 1:10,000 dilution for 2 h at room temperature. After three additional TBST washes, each lasting 10 min, the membranes were incubated with secondary antibodies for 1 h at room temperature. The protein bands were visualized by ECL detection reagents (P10300; NCM Biotech) and quantified using the Image J software, with the bands of interest normalized to β-actin.

### Animal sacrifice and frozen sectioning

The mice were anesthetized with an overdose of sodium pentobarbital (100 mg/kg, intraperitoneal) and subsequently underwent cardiac perfusion with ice-cold PBS. For histopathological investigations, the mice brains were harvested and underwent a 24 h post-fixation in 4% paraformaldehyde (pH 7.4), followed by a gradual dehydration process utilizing sucrose solutions (4 °C, 10–30%, pH 7.4). The mice brains were then rapidly frozen using an OCT embedding compound and serially sectioned with a cryostat microtome (CM1950, Leica, Germany). These serial brain sections (25 µm) were methodically organized into six identical batches for subsequent analyses.

### Immunofluorescence and immunohistochemistry

Immunohistochemistry staining followed previously established protocols^50^. First, for 3,3’-diaminobenzidine (DAB) staining, the sections underwent a 10 min treatment with 3% H_2_O_2_, followed by blocking with 5% bovine serum albumin (BSA) and 0.3% Triton X-100 in PBS at room temperature for 1 h. Then, incubation with a primary antibody was performed at 4 °C for 24 h (mouse anti-TH, 1:1000; T1299, sigma, USA). Sections were then washed in PBS and incubated with a biotinylated secondary antibody (donkey anti-mouse, 1:1000, Vector Labs, USA) for 2 h at room temperature, followed by incubated with the avidin–biotin–peroxidase complex (1:1000, Vector Labs, USA) for 1 h. Finally, the sections were immersed in a DAB solution (Vector Labs, USA) for staining. The primary antibodies used were: anti‐GBA (ab55080; abcam), anti‐α‐synuclein (phospho S129) (ab51253; abcam). For immunofluorescence staining, the slices were incubated with species-appropriate secondary antibodies tagged with Alexa 594 (red, ex/em: 590/ 618 nm) (A21203, Invitrogen) for 2 h. Finally, the sections were stained with DAPI (Southern Biotech) for 5 min. Images were captured by the confocal microscope (ZEISS; LSM 700) and VS200 scan microscope (Olympus).

### Statistical analysis

All data analyses were conducted using Prism 9.0 software and were presented as the mean ± standard error of the mean (SEM). Unpaired Student’s *t*-test was used for comparison between the two groups. Differences among multi-groups were assessed by one-way or two-way ANOVA followed by Bonferroni’s post hoc multiple comparison analysis. A two-tailed *P*-value < 0.05 was deemed as statistically significant.

### Supplementary information

Supplementary Material

Supplementary Video 2 PFF group

Supplementary Video 1 PBS group

## Data Availability

All data generated or analyzed in this study are included in the published article and the supplementary information.
