# Supplementary material for: GBA-AAV mitigates sleep disruptions and motor deficits in mice with REM sleep behavior disorder
Source: NPJ Parkinsons Dis. 2024 Aug 2;10:142. doi: 10.1038/s41531-024-00756-5 (PMC11297138; doi:10.1038/s41531-024-00756-5)
Supplement: Supplementary file 1 — Supplementary Material [file 41531_2024_756_MOESM1_ESM.pdf]

SUPPLEMENTARY FIGURE 1

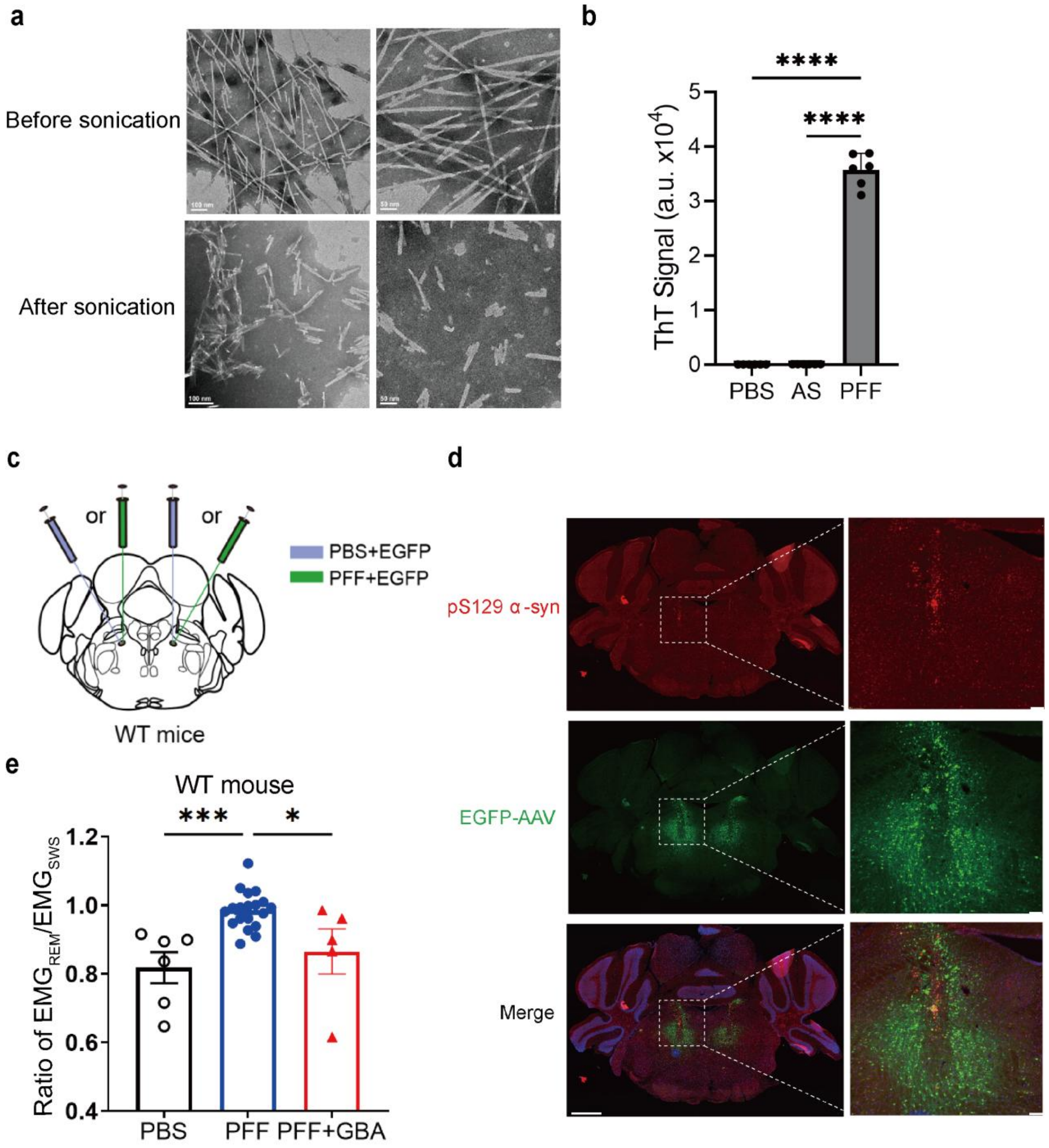

Supplementary Figure 1 Analysis of PFF structures pre- and post-sonication and their induction of RBD-like behaviors in WT Mice. a Transmission electron microscopy images demonstrate alpha-synuclein fibril (PFF) before and after the sonication process. Scale bars, 100 nm (left) and 50 nm (right). **b** The Thioflavin T (ThT) assay is suggested for verifying the existence of beta-sheet structures. ThT fluorescence data show elevated in PFF group comparison to monomers (AS) or PBS (*n* = 6). **c** Diagram outlining the experimental protocol in WT mice, which received either PBS+EGFP or PFF+EGFP treatments. **d** Histological analysis of brain sections

demonstrates the co-localization of pS129 α-synuclein with AAV-EGFP expression. The cell nuclei are stained with DAPI, represented in blue. Scale bars, 1 mm (left) and 100 μm (right). **e** Statistical analysis of the ratio of EMG_{REM}/EMG_{SWS} across various treatments in WT mice. PFF, pre-formed fibril; ThT, thioflavin T; All data are shown as mean ± SEM and all tests were two-sided. One-way ANOVA were used for statistical analysis followed by Bonferroni's multiple comparisons test. $^*P < 0.05$, $^{***}P < 0.001$, $^{****}P < 0.0001$.

SUPPLEMENTARY FIGURE 2

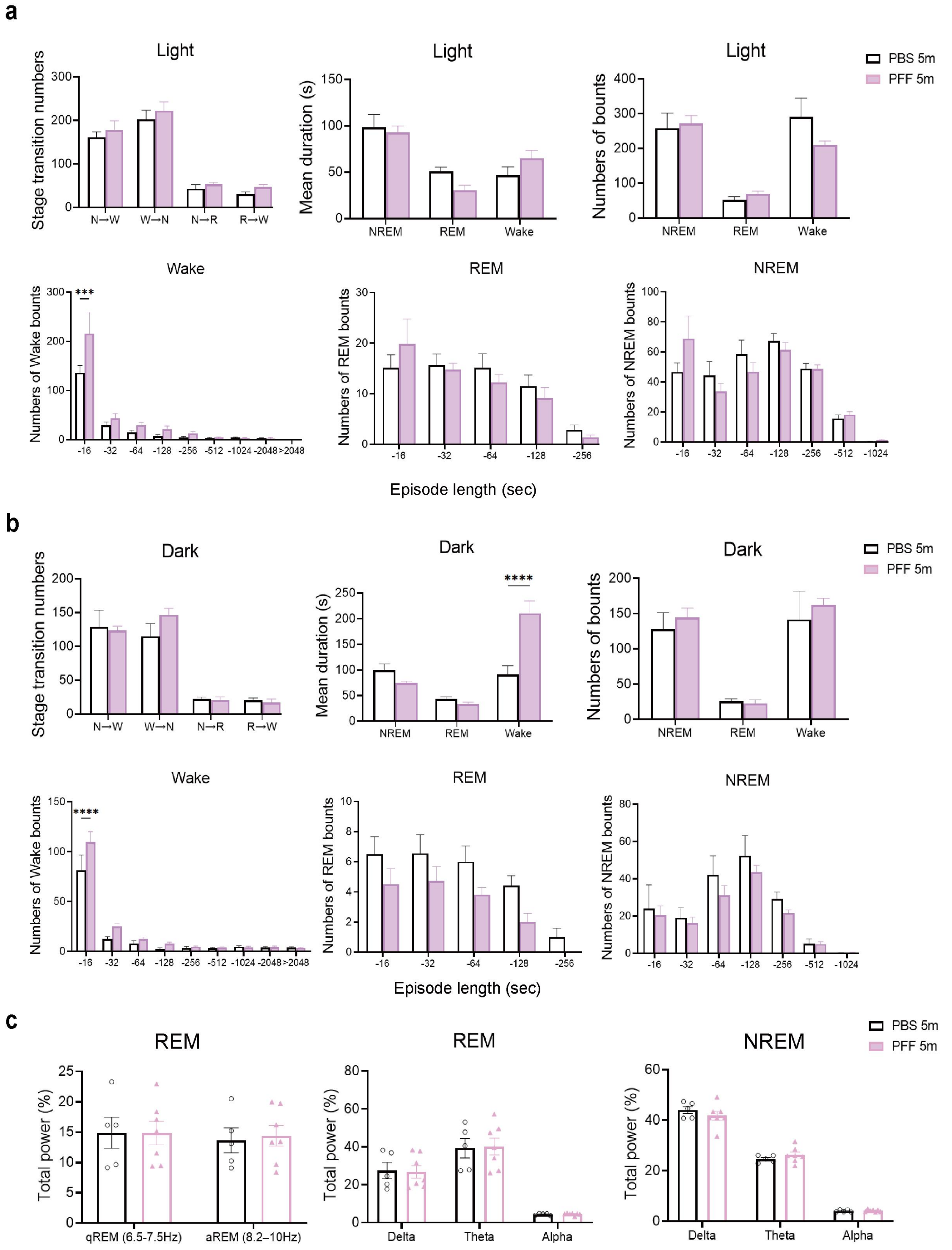

Supplementary Figure 2 Quantitative evaluation of the sleep architecture parameters after PFF infection at five months in WT mice. a-b Assessment of the sleep architecture after PBS or PFF injection at five months in WT mice. Statistical comparison of the mean duration, numbers of bouts, stage transition numbers in Wake, REM, and NREM states during Light and Dark phase. **c** EEG power spectral analysis

during REM and NREM sleep reveals the distribution of frequency bands such as qREM (6.5-7.5Hz), aREM (8.2-10Hz), Delta, Theta, and Alpha. This analysis helps understand neural oscillations and potential changes induced by PFF treatment. The vigilance state transition patterns include N-W, W-N, N-R and R-W. R = rapid eye movement sleep (REM); N = non-rapid eye movement sleep (NREM); W = wake. All data are shown as mean ± SEM and all tests were two-sided (n = 6). Two-way ANOVA were used for statistical analysis followed by Bonferroni's multiple comparisons test. ***$P < 0.001$, ****$P < 0.0001$.

SUPPLEMENTARY FIGURE 3

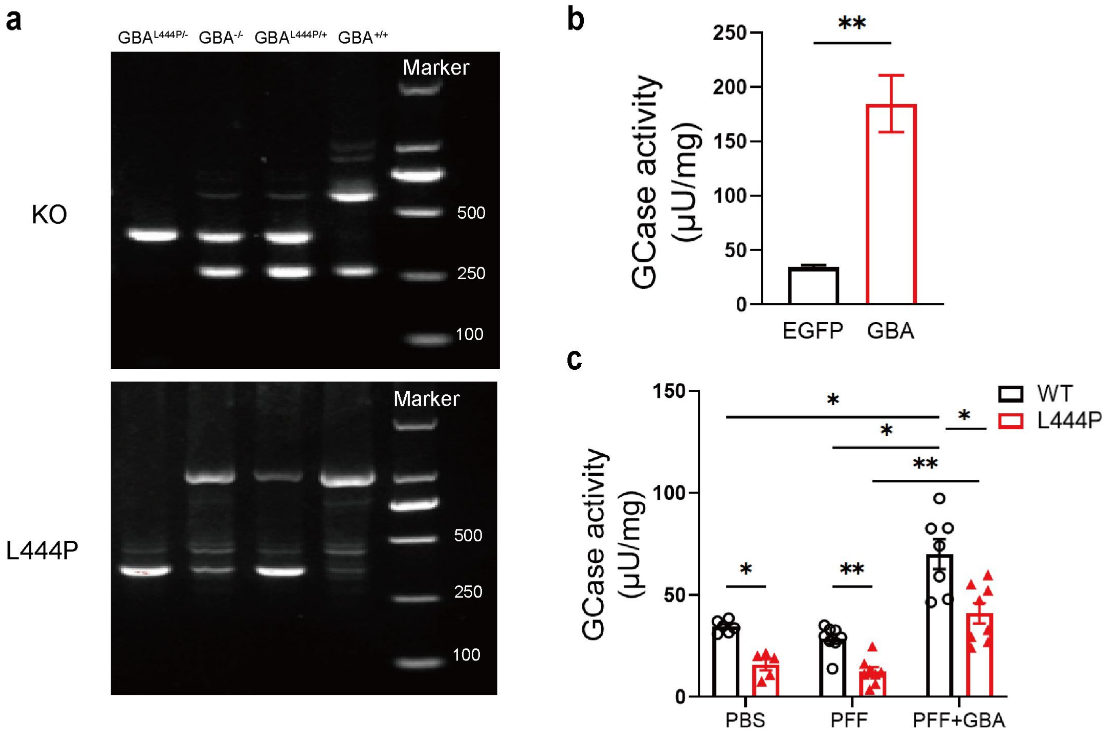

Supplementary Figure 3 Identification of genotypes of GBA L444P mutant and WT mice and changes of GCase activity. a The GBA L444P mutant and WT mice were genotyped by PCR using tail DNA as template. KO primer sequence detected whether the murine-derived GBA gene was knocked out, and L444P Transgene primer sequence detected whether the human-L444P mutation gene was inserted. Homozygote = ~ 400 bp, Heterozygote = ~ 400 bp and 262 bp, Wild type = 262 bp; human L444P Transgene = ~200 bp. **b** Overexpression of GBA-AAV significantly increased GCase activity in the SLD region of WT mice (n = 6). The P values were

calculated using unpaired t tests. **c** Overexpression of GBA-AAV could reverse the decrease of GCase activity caused by PFF, and the GCase activity of L444P mutant mice was significantly decreased compared with WT mice (n = 5-8). All data are shown as mean ± SEM and all tests were two-sided. Two-way ANOVA were used for statistical analysis followed by Bonferroni's multiple comparisons test. *P < 0.05, **P < 0.01.

SUPPLEMENTARY FIGURE 4

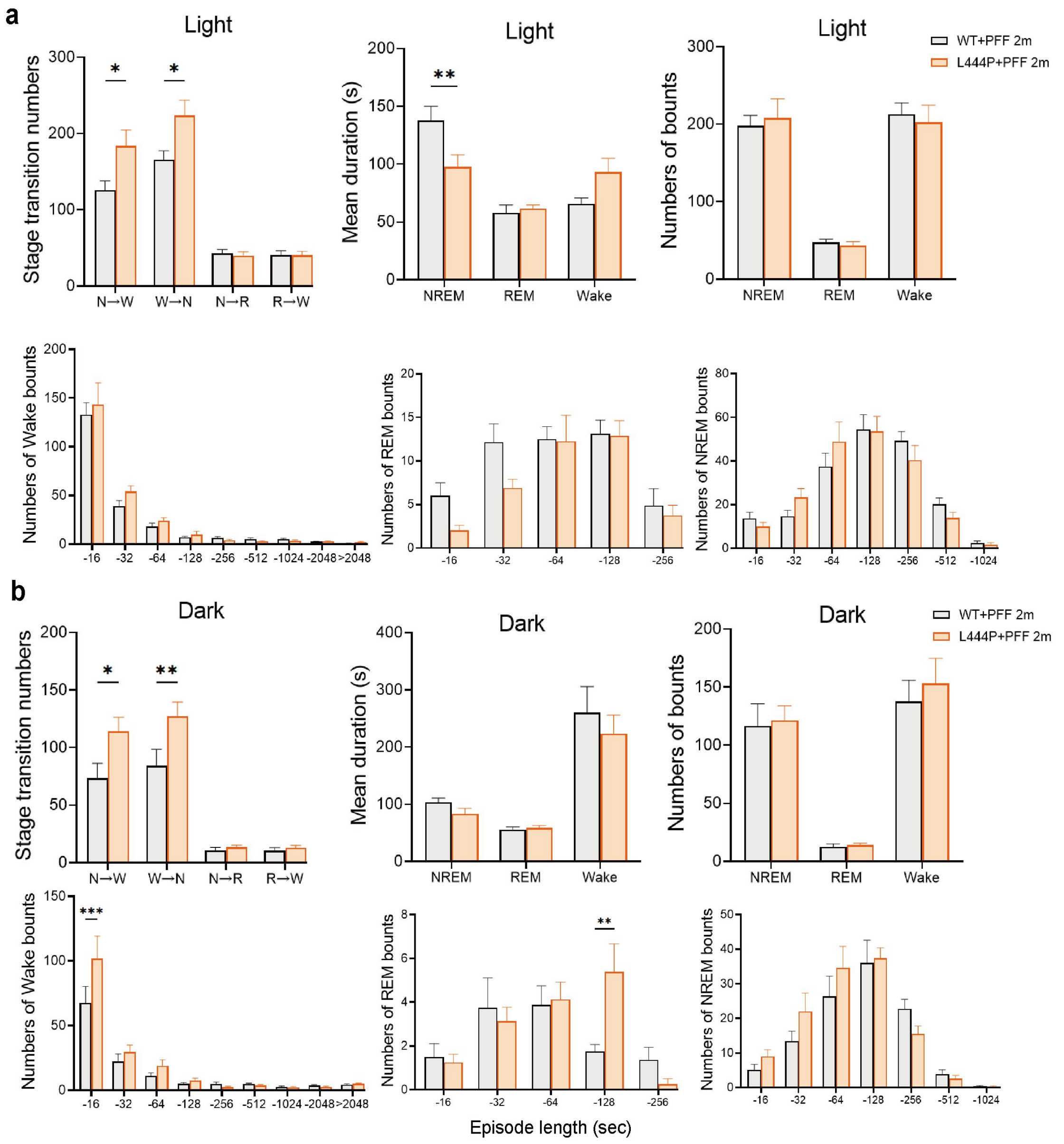

Supplementary Figure 4 Quantitative evaluation of the sleep architecture parameters after infection PFF at two months in WT and L444P mutant mice. **a-b** Assessment of the sleep architecture in WT or L444P mutant mice receiving PFF

injection for two months. Statistical comparison of the mean duration, numbers of bouts, stage transition numbers in Wake, REM, and NREM states during Light and Dark phase (*n* = 6-8). The vigilance state transition patterns include N-W, W-N, N-R and R-W. R = rapid eye movement sleep (REM); N = non-rapid eye movement sleep (NREM); W = wake. All data are shown as mean ± SEM and all tests were two-sided. Two-way ANOVA were used for statistical analysis followed by Bonferroni's multiple comparisons test. *$P < 0.05$, **$P < 0.01$, ***$P < 0.001$.

SUPPLEMENTARY FIGURE 5

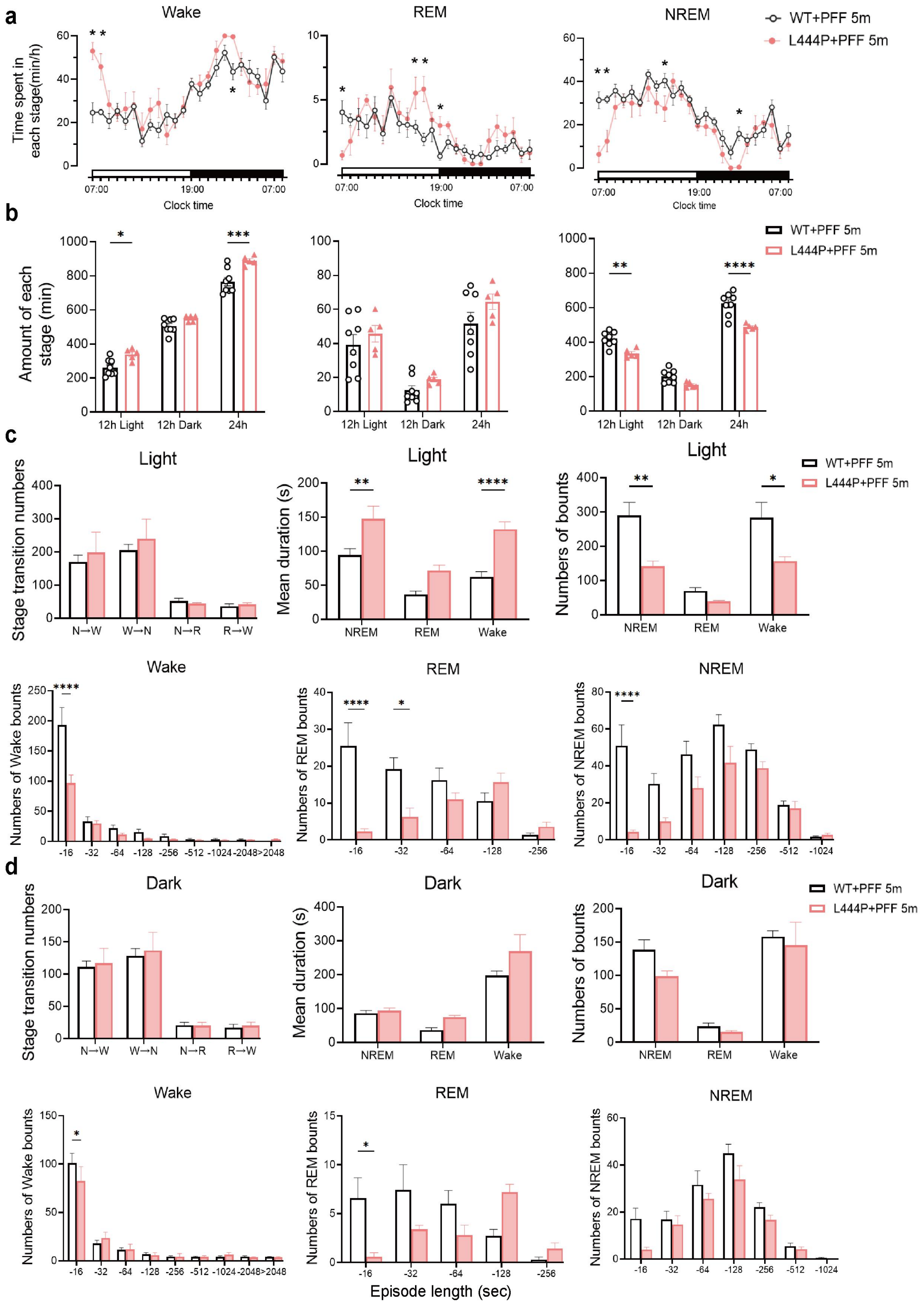

Supplementary Figure 5 Quantitative evaluation of the sleep architecture parameters after infection PFF at five months in WT and L444P mutant mice. **a–b** Time spent in each stage of Wake, REM and NREM sleep after infection PFF at

five months in WT and L444P mutant mice over a 24 h light-dark cycle (n = 8 vs 5). **c-d** Assessment of the sleep architecture in WT or L444P mutant mice receiving PFF injection for five months. Statistical comparison of the mean duration, numbers of bouts, stage transition numbers in Wake, REM, and NREM states during Light and Dark phase (*n* = 8 vs 5). The vigilance state transition patterns include N-W, W-N, N-R and R-W. R = rapid eye movement sleep (REM); N = non-rapid eye movement sleep (NREM); W = wake. All data are shown as mean ± SEM and all tests were two-sided. Two-way ANOVA were used for statistical analysis followed by Bonferroni's multiple comparisons test. $*P < 0.05$, $**P < 0.01$, $****P < 0.0001$.

79 **SUPPLEMENTARY FIGURE 6**

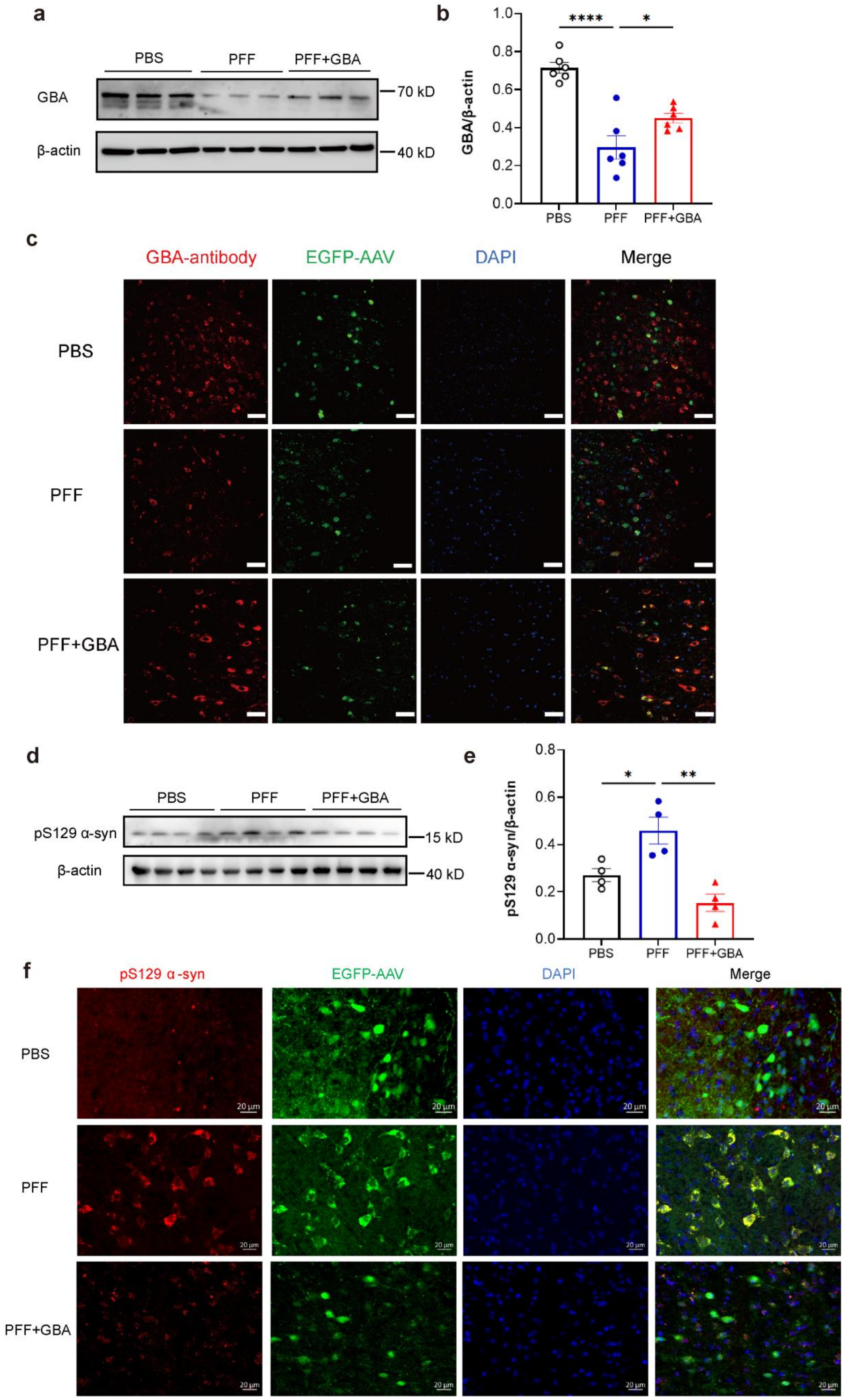

80

Supplementary Figure 6 GBA-AAV over-expression increased the GBA protein level and reduced the aggregation of PFF-induced serine 129 phosphorylated α-synuclein (pS129 α-syn). a-b Immunoblotting and quantification reveal increased GBA protein levels between the PBS, PFF and PFF + GBA mouse groups (n = 6). **c** Immunofluorescence highlights GBA in the SLD region across the PBS, PFF and PFF + GBA mouse groups (n = 4). Scale bars, 50μm. **d-e** Immunoblotting and quantification of pS129 α-syn between the PBS, PFF and PFF + GBA mouse groups (n = 4). **f** Immunofluorescence staining of pS129 α-syn in the SLD region between the PBS, PFF and PFF + GBA mouse groups (n = 4). Scale bars, 20μm. Data are shown as mean ± SEM. One-way ANOVA followed by Bonferroni's post-tests. All tests were two-sided. *$P < 0.05$, **$P < 0.01$, ****$P < 0.0001$.

SUPPLEMENTARY FIGURE 7

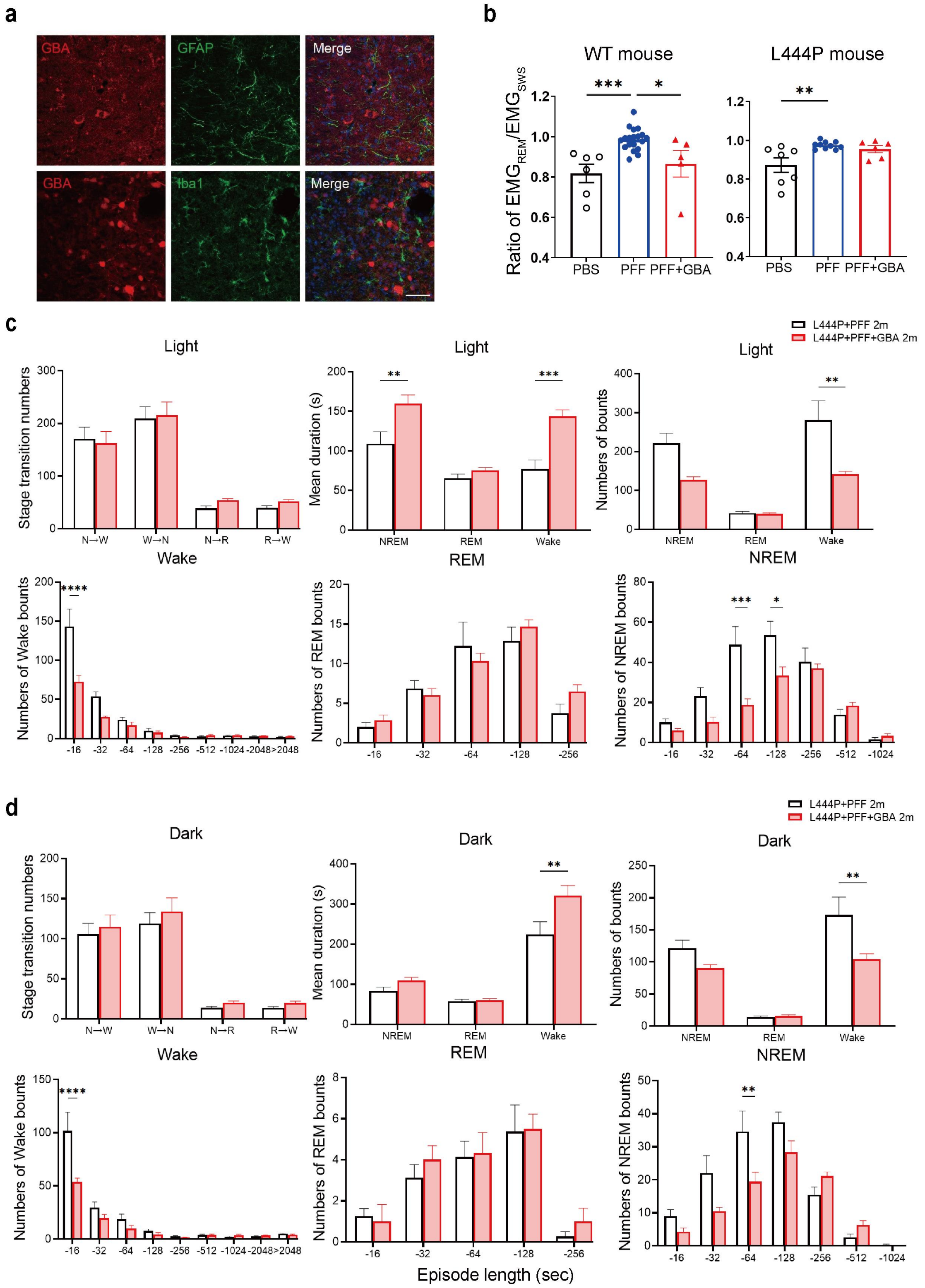

Supplementary Figure 7 Effects of GBA-AAV treatments on muscle tension during REM sleep and total sleep patterns after PFF injection for two months in L444P mutant mice. a Immunofluorescence image showed that GBA protein was not

colocalized with GFAP (marking glial cells) or Iba1 (marking microglial cells). Scale bars, 50μm. **b** Statistical analysis of the ratio of EMG_{REM}/EMG_{SWS} between WT or L444P mutant mice in the PBS, PFF and PFF + GBA-AAV groups after two months (n = 6-14). **c-d** Assessment of the sleep architecture in mice receiving PFF or PFF + GBA-AAV injection in L444P mutant mice. Statistical comparison of the mean duration, numbers of bouts, stage transition numbers in Wake, REM, and NREM states during Light and Dark phase (n = 6). The vigilance state transition patterns include N-W, W-N, N-R and R-W. R = rapid eye movement sleep (REM); N = non-rapid eye movement sleep (NREM); W = wake. All data are shown as mean ± SEM. Two-way ANOVA were used for statistical analysis followed by Bonferroni's multiple comparisons test. *$P < 0.05$, **$P < 0.01$, ***$P < 0.001$, ****$P < 0.0001$.

Uncropped WB Supplementary Fig. 6

a

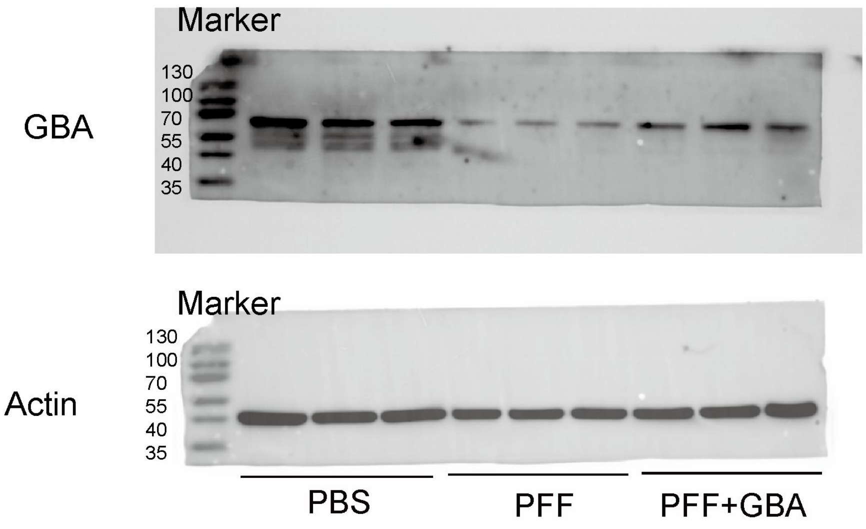

d

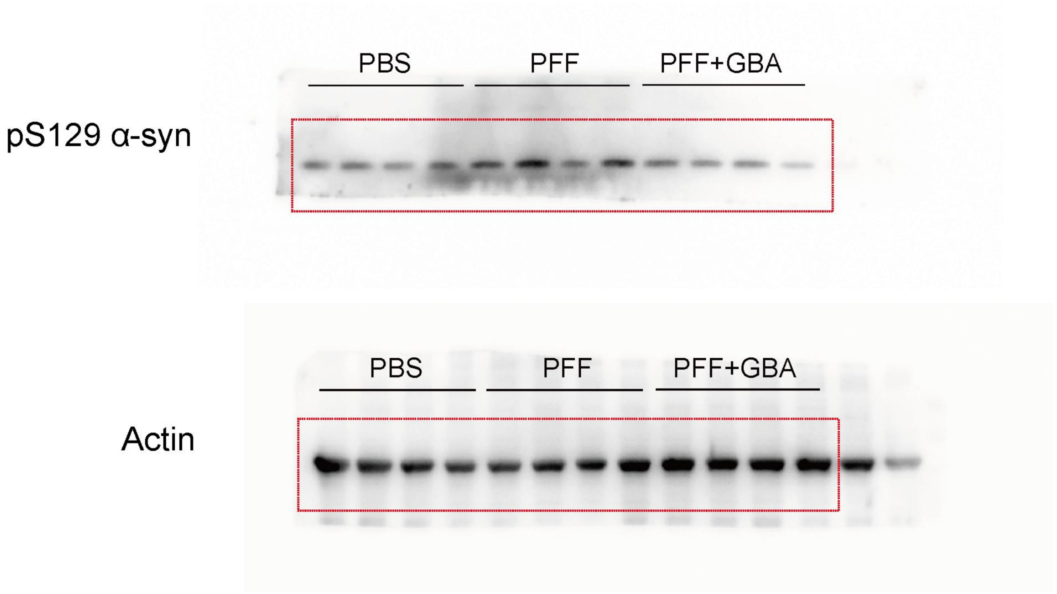

Supplementary Video Legends

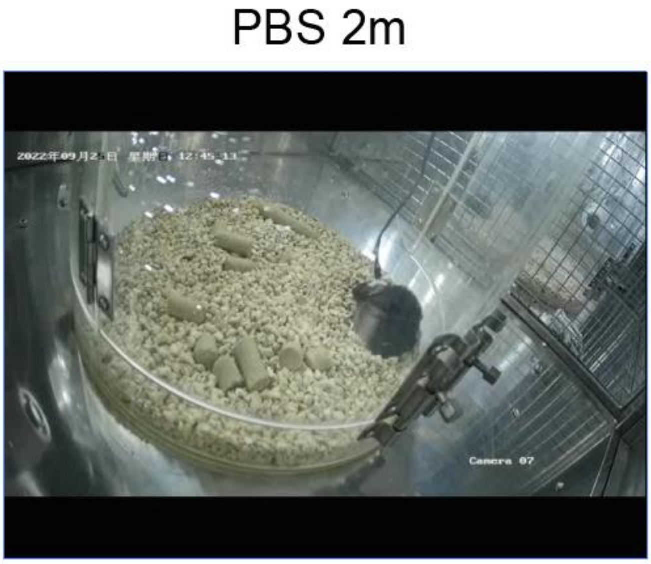

Supplementary video 1 PBS injection into the SLD nucleus at two months in WT mice. Throughout the REM episode, the mouse did not show RBD-like behavior in the PBS group.

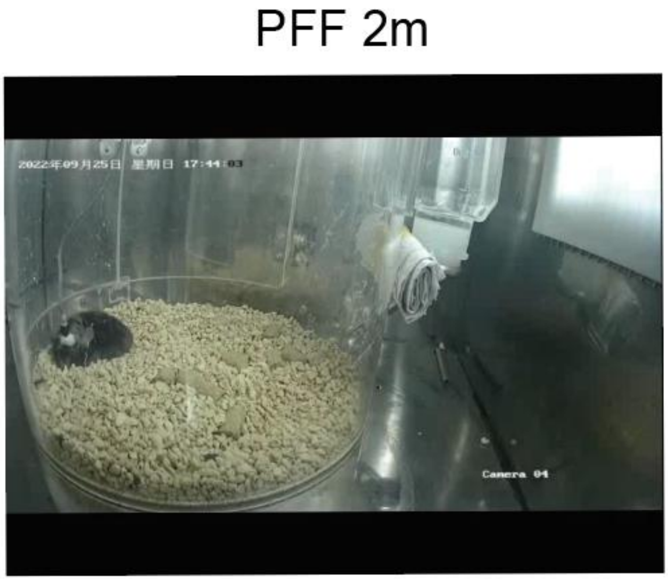

Supplementary video 2 PFF injection into the SLD nucleus at two months in WT mice. Throughout the REM episode, the mouse showed milder movements, including limb jerking, shaking or twisting the body for a few moments, or even exhibited violent jumping up or running out in the PFF group.
